# Novel Universal Bond Containing Bioactive Monomer Promotes Odontoblast Differentiation In Vitro

**DOI:** 10.3390/jfb14100506

**Published:** 2023-10-10

**Authors:** Yaxin Rao, Youjing Qiu, Bayarchimeg Altankhishig, Yasuhiro Matsuda, Md Riasat Hasan, Takashi Saito

**Affiliations:** 1 Division of Clinical Cariology and Endodontology, Department of Oral Rehabilitation, School of Dentistry, Health Sciences University of Hokkaido, Tobetsu 061-0293, Hokkaido, Japan; artionrao@hoku-iryo-u.ac.jp (Y.R.); bayarchimeg@hoku-iryo-u.ac.jp (B.A.); ymatsuda@hoku-iryo-u.ac.jp (Y.M.); riasat@hoku-iryo-u.ac.jp (M.R.H.); 2 Stomatological Hospital of Xiamen Medical College and Xiamen Key Laboratory of Stomatological Disease Diagnosis and Treatment, Xiamen 361008, China; qiuyoujing@gmail.com

**Keywords:** bioactive monomer, CMET, universal bond, adhesive materials, odontoblast differentiation

## Abstract

The development of multifunctional materials has been expected in dentistry. This study investigated the effects of a novel universal bond containing a bioactive monomer, calcium 4-methacryloxyethyl trimellitic acid (CMET), on odontoblast differentiation in vitro. Eluates from bioactive universal bond with CMET (BA (+), BA bond), bioactive universal bond without CMET (BA (−)), and Scotchbond Universal Plus adhesive (SC, 3M ESPE, USA) were added to the culture medium of the rat odontoblast-like cell line MDPC-23. Then, cell proliferation, differentiation, and mineralization were examined. Statistical analyses were performed using a one-way ANOVA and Tukey’s HSDtest. The cell counting kit-8 assay and alkaline phosphatase (ALP) assay showed that cell proliferation and ALP were significantly higher in the 0.5% BA (+) group than in the other groups. In a real-time reverse-transcription polymerase chain reaction, mRNA expression of the odontogenic markers, dentin sialophosphoprotein (DSPP) and dentin matrix protein-1 (DMP-1), was significantly higher in the 0.5% BA (+) group than in the BA (−) and SC groups. Calcific nodule formation in MDPC-23 cells was accelerated in the BA (+) group in a dose-dependent manner (*p* < 0.01); however, no such effect was observed in the BA (−) and SC groups. Thus, the BA bond shows excellent potential for dentin regeneration.

## 1. Introduction

Dental caries is an infectious microbiological disease of the teeth. It starts with the demineralization process of the calcified tissues through acidic products from the bacterial fermentation of dietary carbohydrates, followed by localized dissolution and destruction of the tooth substrate [1,2]. It progresses to deeper portions of the tooth, eventually invading the dental pulp tissue and causing inflammation and pain. Thus, to avoid the occurrence of pulp inflammation, the removal of the infected tooth structure and then dental restoration or dental filling is performed using dental materials such as composite resin [3,4], ceramics, and gold alloy to restore the lost tooth substrate’s function, integrity, and morphology.

In 2000, FDI World Dental Federation (FDI) proposed an innovative dental caries management policy called Minimal Intervention (MI) and revised it to Minimal Intervention Dentistry (MID) in 2006, which has changed the preventive management and treatment of dental caries drastically [5,6]. With the spread of the MID concept, preserving as much of the tooth substance and dental pulp tissue as possible in treating dental caries, such as root surface caries and deep-seated caries, is recommended. To practice MID effectively, the development of multifunctional adhesive materials with the ability to induce remineralization, reparative dentin formation, and antibacterial and anti-biofilm formation activity has been expected. Dental material must possess biocompatibility, as it is highly related to the clinical success rate of vital pulp therapy (VPT), particularly direct pulp capping [7]. Although calcium hydroxide [Ca(OH)_2_] is widely used and is considered the “gold standard” material for VPT [8], it is also associated with limitations such as cytotoxicity and a lack of adhesiveness on the dentin and dental pulp tissues [9,10,11,12]. The biocompatibility and mechanical properties of mineral trioxide aggregate (MTA) are superior to those of Ca(OH)_2_ [13,14]. However, it is associated with drawbacks such as discoloration [15] and poor adhesion to the existing dental adhesive systems [16,17], which affect the clinical outcomes and convenience of restorative procedures. Biodentine, a tricalcium silicate-based cement, has been introduced as an alternative to MTA and has the advantages of a faster setting time, lesser discoloration, and improved adhesive properties [18,19]. However, Biodentine is still far inferior to regular dental adhesive systems in its adhesion to tooth substrate, leaving the possibility of secondary infection to the dental pulp tissue later on. Thus, bioactive functional materials possessing adhesion abilities are ideal for dentin regeneration in the VPT.

The widespread adoption of the MID concept has led to dramatic advances in adhesive materials development. Many dental adhesive systems have developed from a perspective of mainly adhesion and maneuverability so far. On the other hand, material development focused on functionality has also been initiated [20]; antibacterial monomers, 12-methacryloyloxydodecylpyridinium bromide (MDPB) and 2-methacryloyloxyethyl phosphorylcholine (MPC) [21,22], and surface pre-reacted glass ionomers (S-PRG) showing mineral-inducing activity [23] are good examples. Based on the MID concept, our research group aims to develop multi-purpose materials that elicit intensive self-healing abilities in carious dentin and exposed pulp tissue due to deep-seated caries. 

**Figure 1:**
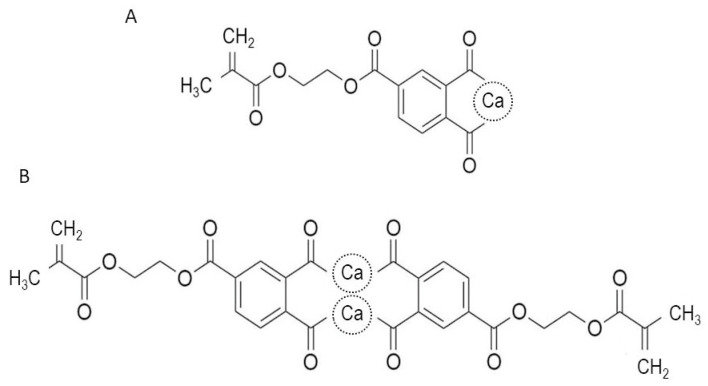
Chemical structure of CMET. (**A**) Monomer, (**B**) dimer. CMET, calcium 4-methacryloxyethyl trimellitic acid.

A previous study has reported that 4-methacryloxyethyl trimellitic acid (4-MET) has a vital role in promoting the infiltration of monomers into the peripheries of enamel prisms, resulting in strong adhesion between the dental adhesive and etched enamel surface [24]. We recently developed CMET, the calcium salt of 4-MET, as a bioactive monomer to add value to 4-MET. CMET was created by replacing hydrogen ions in the two carboxyl groups with calcium ions in 4-MET to neutralize it. It exists as a monomer and dimer [25] (Figure 1). CMET induced the remineralization of demineralized dentin in metastable mineralizing solutions in vitro and increased the mechanical properties of resin-based coating materials [25]. It inhibited biofilm formation by *Streptococcus mutans,* which is one of the cariogenic bacteria [26]. Moreover, it exhibited lower cytotoxicity, in addition to inducing intensive differentiation of odontoblast-like cells and dental pulp stem cells in vitro and reparative dentin formation in rats [27,28]. Therefore, CMET was demonstrated as a multifunctional bioactive monomer. We prototyped a novel universal bond with the bioactive monomer CMET based on these findings. 

This study aimed to investigate the effects of the novel universal bond with the bioactive monomer CMET on odontoblast differentiation in vitro and elaborate its multifunctional properties.

## 2. Materials and Methods

### 2.1. Preparation of Aqueous Solutions of the Materials

Bioactive universal bond with CMET (BA (+), BA bond), bioactive universal bond without CMET (BA (−)), and Scotchbond Universal Plus adhesive (SC, 3M ESPE, USA), were used in this study (Table 1). Discs made of the materials were fabricated using a silicone mold and immersed in distilled water at 37 °C for seven days. Subsequently, the eluate of BA (+) was added into the culture medium at 0, 0.1, 0.5, 1, and 5% (*v*/*v*), and the eluates of BA (−) and SC were added into the culture medium at 0.5% (*v*/*v*).

### 2.2. Cell Culture

The cell culture and analyses were performed following the methods employed in the previous studies [27]. The rat odontoblast-like cell line, MDPC-23, was obtained (Professor Nör; University of Michigan). The cells were seeded into 96-well culture plates at 2.0 × 10^3^ cells/well and into 12-well plates at 2.0 × 10^4^ cells/well (Corning, NY, USA) and incubated in growth medium containing Dulbecco’s modified Eagle’s medium (Sigma-Aldrich, St. Louis, MO, USA) supplemented with 5% fetal bovine serum (FBS, Gibco, Grand Island, NY, USA). The mineralization medium was prepared by adding glycerol-2-phosphate disodium salt n-hydrate (10 mmol/L; Wako, Osaka, Japan) and L-ascorbic acid phosphate magnesium salt n-hydrate (50 µg/mL; Wako) into the growth medium and was used for the culture on day 5 after reaching confluence. The cells were cultured in a humidified 5% carbon dioxide-balanced air incubator at 37 °C. Cell passage 27 was used for the experiments.

### 2.3. Cell Proliferation

The cells (2.0 × 10^3^ cells/well) were seeded into non-tissue culture-treated 96-well plates and cultured in the growth medium. BA (+) (0.1%, 0.5%, 1%, and 5% (*v*/*v*)), 0.5% BA (−) (*v*/*v*), and 0.5% SC (*v*/*v*) were added to the medium on day 1. On day 5, cell viability was measured using cell counting kit-8 (CCK-8; Dojindo, Kumamoto, Japan). The CCK-8 reagent was added to the wells (10 µL/well) and incubated for 90 min. The absorbance at 450 nm was measured using a microplate reader (Bio-Rad, Hercules, CA, USA).

### 2.4. Alkaline Phosphatase Activity

The cells (2.0 × 10^4^ cells/well) were cultured on non-tissue culture-treated 12-well plates with the growth medium for five days. Then, BA (+) (0.1%, 0.5%, 1%, and 5% (*v*/*v*)), 0.5% BA (−) (*v*/*v*), and 0.5% SC (*v*/*v*) were added to the mineralization medium. An alkaline phosphatase (ALP) activity assay was performed on day 7 using the LabAssay^TM^ALP kit (WAKO) to detect early-stage cell differentiation toward the odontogenic lineage. Protein quantification was performed using the Thermo Scientific^TM^ Pierce^TM^ BCA protein assay kit (Thermo Fisher Scientific) according to the method previously reported [27].

### 2.5. Quantitative Real-Time Reverse-Transcription Polymerase Chain Reaction 

The cells (2.0 × 10^4^ cells/well) were cultured on non-tissue culture-treated 12-well plates with the growth medium for five days. Then, BA (+) (0.1%, 0.5%, 1%, and 5% (*v*/*v*)), 0.5% BA (−) (*v*/*v*), and 0.5% SC (*v*/*v*) were added to the mineralization medium. The total RNA was extracted using TRIzol^®^ reagent (Invitrogen, Carlsbad, CA, USA) on day 7. Moloney murine leukemia virus (M-MLV) reverse transcriptase (Thermo Fisher Scientific) was used to reverse transcribe RNA (1 µg) to complementary DNA in a 20 µL reaction system (cDNA: 1 µL; forward primer: 1 µL; backward primer: 1 µL; FastStart Essential DNA Green Master PCR grade H_2_O (Roche, Basel, Switzerland): 7 µL; FastStart Essential DNA Green Master 2× conc. (Roche): 10 µL) according to the method previously reported [29]. The mRNA expressions of dentin sialophosphoprotein (DSPP) and dentin matrix protein-1 (DMP-1) as dentinogenesis-related genes were quantified using quantitative real-time reverse-transcription polymerase chain reaction (RT-PCR). Quantitative real-time RT-PCR was performed using a LightCycler^®^ Nano (Roche) according to the manufacturer’s instructions. Table 2 presents the primer sequences and PCR conditions for the LightCycler^®^ Nano. The comparative 2^−ΔΔCT^ method was applied to determine the relative changes in the gene expression compared to the reference housekeeping gene β-actin. 

### 2.6. Mineralization

The cells (2.0 × 10^4^ cells/well) were cultured on non-tissue culture-treated 12-well plates with the growth medium for five days. Then, BA (+) (0.1%, 0.5%, 1%, and 5% (*v*/*v*)), 0.5% BA (−) (*v*/*v*), and 0.5% SC (*v*/*v*) were added to the mineralization medium. Alizarin Red S (ARS) staining was performed on days 8 and 9 to detect calcium deposition in the extracellular matrices [27]. The cells were washed with phosphate-buffered saline (PBS; pH 7.4, Gibco), fixed in a 10% formalin neutral buffer solution (Wako) for 20 min at room temperature, rinsed with PBS, and stained with ARS solution (1%, pH 4.1; Wako). The staining solution was removed after 10 min. The cells were washed three times with distilled water and then with PBS to remove non-specifically bound staining. Images were acquired using a digital imaging system (Funakoshi, Tokyo, Japan). Cetylpyridinium chloride (CPC) was used to measure the staining intensity quantitatively. After staining with ARS, 10% CPC (Sigma-Aldrich) aqueous solution (*w*/*v*), was added to the wells and incubated for 1 h at 37 °C. Subsequently, the cells were detained with 10% CPC solution for 20 min at room temperature. Then, the ARS concentration was determined by measuring the absorbance at a wavelength of 570 nm.

### 2.7. Statistical Analyses

All experiments were performed in triplicate. The data obtained from the experiments are expressed as means ± standard deviations. The statistical significance of the differences among the data was analyzed using one-way analyses of variance (ANOVA) and Tukey’s honest significant difference (HSD) test. Differences were considered significant for *p <* 0.01. 

## 3. Results

### 3.1. Cell Proliferation

**Figure 2:**
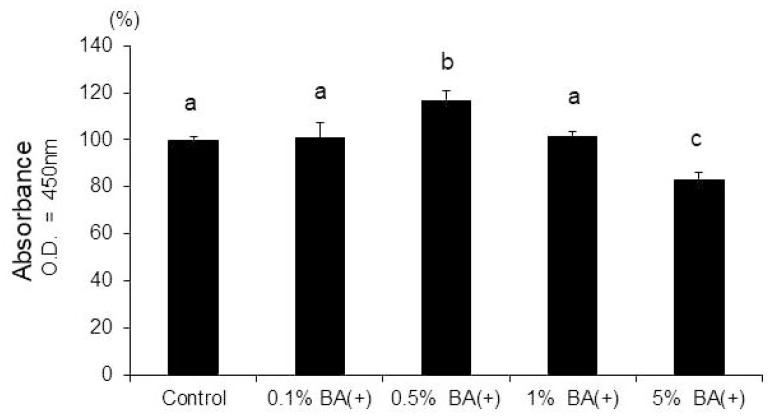
CCK-8 cell viability assay. The MDPC-23 cells were seeded in non-tissue culture-treated 96-well plates at 2.0 × 10^3^ cells/well. BA (+) (0.1%, 0.5%, 1%, and 5% (*v*/*v*)) was added to the growth medium from day 1. The CCK-8 assay was performed on day 5 (*n* = 6). Different lowercase letters indicate significant differences (*p* < 0.01). CCK-8, cell counting kit-8.

**Figure 3:**
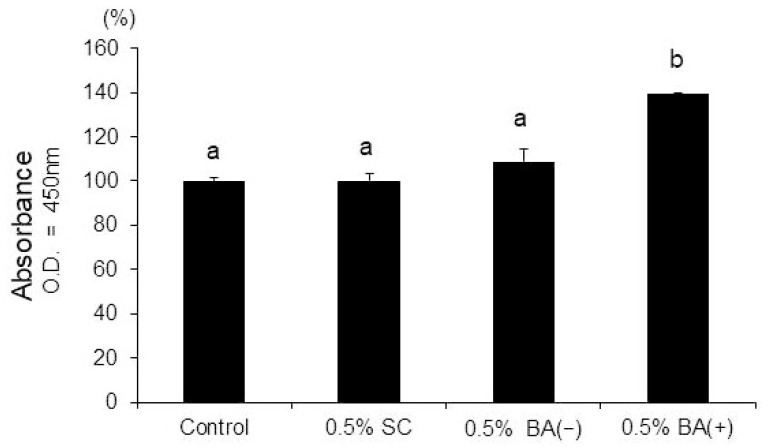
CCK-8 cell viability assay. The MDPC-23 cells were seeded in non-tissue culture-treated 96-well plates at 2.0 × 10^3^ cells/well. Subsequently, 0.5% (*v*/*v*) of each material was added to the growth medium separately from day 1. The CCK-8 assay was performed on day 5 (*n* = 6). Different lowercase letters indicate significant differences (*p* < 0.01).

In the CCK-8 assay, a comparison of the different concentrations of BA (+) revealed that the 0.5% BA (+) group stimulated the proliferation of the MDPC-23 cells (*p* < 0.01) (Figure 2). However, the 0.5% BA (−) and SC groups did not affect the proliferation of the MDPC-23 cells (*p* > 0.01) (Figure 3).

### 3.2. ALP Activity

**Figure 4:**
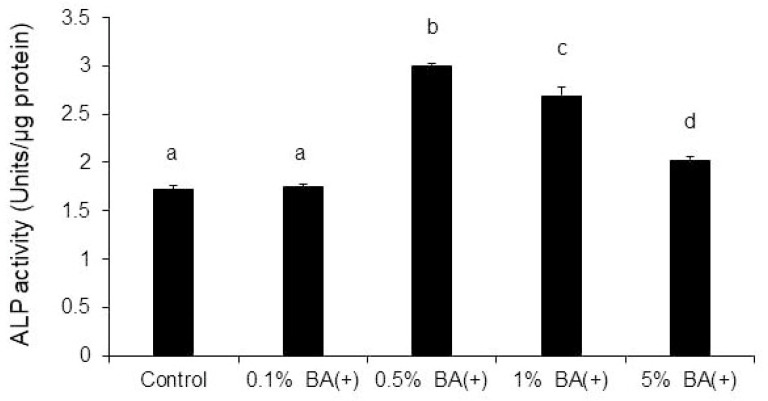
ALP activity. The MDPC-23 cells (2.0 × 10^4^ cells/well) were cultured on non-tissue culture-treated 12-well plates with BA (+) (0.1%, 0.5%, 1%, and 5% (*v*/*v*)) for seven days. The ALP activity was assessed subsequently. Different lowercase letters indicate significant differences (*p* < 0.01). ALP, alkaline phosphatase.

**Figure 5:**
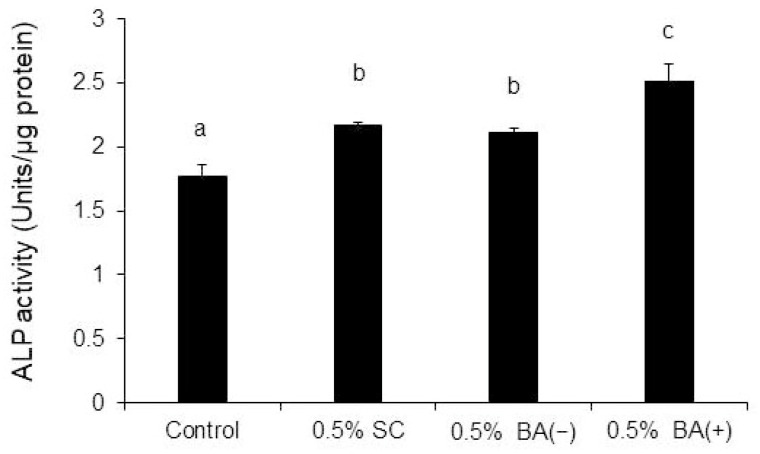
ALP activity. MDPC-23 cells (2.0 × 10^4^ cells/well) were seeded into non-tissue culture-treated 12-well plates with 0.5% (*v*/*v*) of each material for seven days. The ALP activity was assessed subsequently. Different lowercase letters indicate significant differences (*p* < 0.01).

A comparison of the different concentrations of BA (+) revealed that compared with the control group, the ALP activity was significantly promoted on day 7 in all groups except for the 0.1% BA (+) group. The maximum effect was observed in the 0.5% BA (+) group (Figure 4). Compared with the BA (−) and SC groups, the maximum effect was observed in the 0.5% BA (+) group (Figure 5).

### 3.3. Quantitative Real-Time RT-PCR

**Figure 6:**
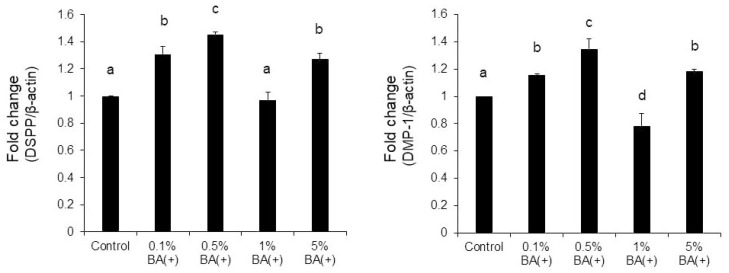
Quantitative real-time RT-PCR. The MDPC-23 cells were cultured on non-tissue culture-treated 12-well plates with BA (+) (0.1%, 0.5%, 1%, and 5% (*v*/*v*)) for seven days. The mRNA expressions of DSPP and DMP-1 were quantified using LightCycler^®^ Nano. Different lowercase letters indicate significant differences (*p* < 0.01). DMP-1, dentin matrix protein-1; DSPP, dentin sialophosphoprotein; RT-PCR, reverse-transcription polymerase chain reaction.

**Figure 7:**
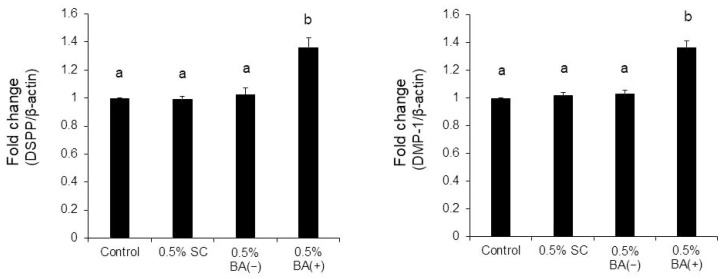
Quantitative real-time RT-PCR. The MDPC-23 cells were cultured on non-tissue culture-treated 12-well plates with 0.5% (*v*/*v*) of each material for seven days as indicated. The mRNA expressions of DSPP and DMP-1 were quantified using LightCycler^®^ Nano. Different lowercase letters indicate significant differences (*p* < 0.01).

Quantitative real-time RT-PCR was conducted to examine the effects of the new bonding system on odontogenic differentiation. It was found that compared with the other concentrations of BA (+), 0.5% BA (+) significantly enhanced the mRNA expression of the two critical dentinogenesis-related markers, DSPP and DMP-1 (Figure 6). Compared with the 0.5% BA (−) and SC groups, the 0.5% BA (+) group showed significantly increased mRNA levels of DSPP and DMP-1 (*p* < 0.01) (Figure 7).

### 3.4. Mineralization

**Figure 8:**
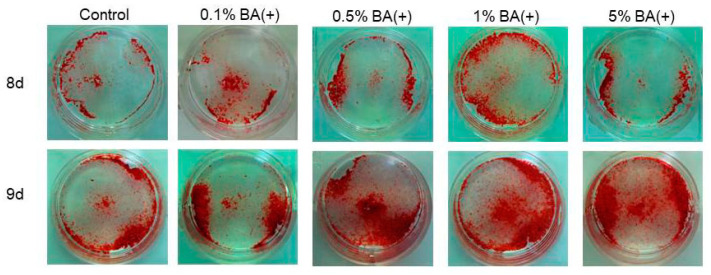
Photographs depicting calcific nodule formation at different concentrations of BA (+) (0.1%, 0.5%, 1%, and 5% (*v*/*v*)) added to MDPC-23 cell culture after eight and nine days.

**Figure 9:**
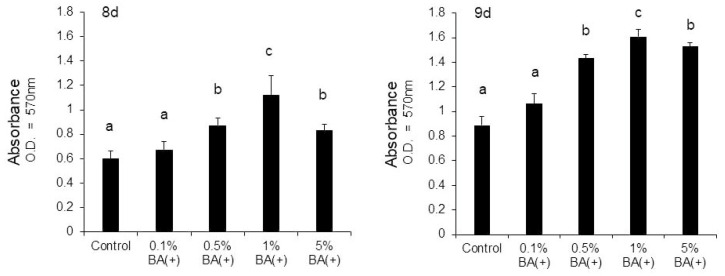
Mineralization induced by different concentrations of BA (+) (0.1%, 0.5%, 1%, and 5% (*v*/*v*)) added to the MDPC-23 cell culture after eight and nine days. CPC quantification of the staining intensity of the mineralized product was performed. Different lowercase letters indicate significant differences (*p* < 0.01). CPC, cetylpyridinium chloride.

**Figure 10:**
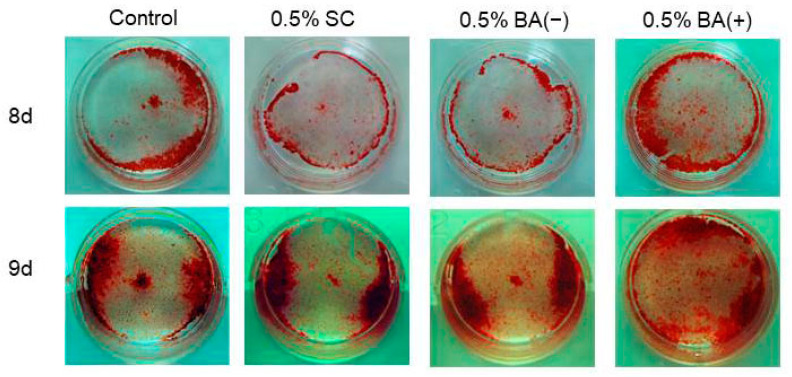
Photographs of calcific nodules formed by 0.5% (*v*/*v*) of each material added in MDPC-23 cell culture after eight and nine days.

**Figure 11:**
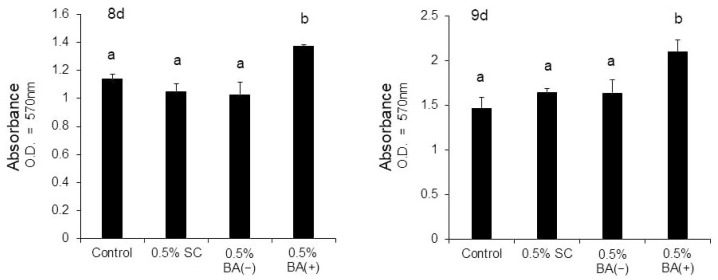
Mineralization induced by the addition of 0.5% (*v*/*v*) of each material to the MDPC-23 cell cultures after eight and nine days. CPC quantification of the staining intensity of the mineralized product was performed. Different lowercase letters indicate significant differences (*p* < 0.01).

The cells were stained with ARS on days 8 and 9 to evaluate the effects of the new bonding system on the expression of the mature odontoblast phenotype. It was found that the new bonding system, the BA (+) group, significantly facilitated calcific nodule formation in the MDPC-23 cells in a dose-dependent manner under the mineralization-inducing condition. CPC quantification confirmed a significant increase in mineralization in the 0.5%, 1%, and 5% BA (+) groups compared with the control group (*p* < 0.01) (Figure 8 and Figure 9). Furthermore, an increase in the calcific nodule formation was observed in the 0.5% BA (+) group compared with the 0.5% BA (−) and SC groups on days 8 and 9 (*p* < 0.01) (Figure 10 and Figure 11).

## 4. Discussion

As the aging of society is recently progressing in many developed countries of the world, the demand of the public on medical sciences has shifted from “longevity” to “enhanced quality of life (QOL)”, due to which regenerative medicine for various organs and tissues has been gaining attraction. In particular, the regeneration of hard tissues is one of the most significant goals related to the enhancement of QOL. In the field of dentistry, the development of new technologies for the restoration of damaged oral tissues due to caries/periodontal disease-related defects in dental/alveolar bone tissues using materials capable of inducing dental/alveolar bone tissue regeneration, instead of the conventional method of replacing defects with artificial substitutes, is required. Treatment methods for periodontal tissue regeneration based on cell biological concepts have been established in the field of periodontology using cell biological agents, including an enamel matrix derivative (Emdogain^®^: Kaken Pharmaceutical, Tokyo, Japan) [30] and basic fibroblast growth factor (FGF-2, REGROTH^®^: Strauman Japan, Tokyo, Japan) [31], as well as changing the passive treatment of periodontal diseases to a proactive therapeutic method. Regarding dental caries treatment, the significance of the dentin–pulp complex is reconfirmed in terms of the goal of long-term preservation of the teeth and their functionality in the oral cavity. FDI proposed a new dental caries management policy called MI in 2000 and revised it to MID in 2006, which has changed the preventive management and treatment of dental caries drastically [5,6]. At the same time, the deep-seated caries treatment policy has also followed the MID concept [32], resulting in the emergence of dentin–pulp regeneration that maximizes self-restoration capacity to repair and induce tissue regeneration. This study successfully showed the development of excellent bioactive material to regenerate tooth substrate by inducing odontoblast differentiation. Moreover, this is the first report regarding multifunctional adhesive material for treatment of caries based on the MID concept. 

Composite resins are one of the most commonly used restorative materials in modern dentistry. The success of composite restorations relies on appropriate bonding techniques and materials [33,34]. The hybrid layer, which is formed when resin-based dental materials are bonded to demineralized dentin, is critical for the longevity and stability of adhesive restorations. The hybrid layer consists of partially demineralized collagen fibers and resin tags that extend into the dentinal tubules, which provide micromechanical retention and resistance to stress from mastication and occlusion [35]. Incomplete impregnation of resin into the dentin can result in vulnerable areas in the hybrid layer, leading to collagen degradation and compromised bonding strength between resin and dentin, resulting in failure of the material from the tooth [36]. Therefore, adhesive systems with the potential to induce remineralization would be beneficial, as they can promote the formation of hydroxyapatite crystals and stabilize the hybrid layer. This function can help prevent water penetration and collagen degradation, thereby improving the long-term performance of adhesive restorations. The existence of collagen fibrils in the hybrid layer can be considered to represent the same state as carious dentin, where collagen fibrils become exposed due to demineralization of dentin. Our previous studies revealed that CMET can induce dentin remineralization in vitro [25] and inhibit biofilm formation by *S. mutans* [26]. Moreover, it can induce intensive differentiation of odontoblast-like cells and dental pulp stem cells and reparative dentin formation in rats [27,28]. Thus, CMET can function as a bioactive adhesive monomer. The results of this study demonstrated that the BA bond, a bonding agent with CMET, exhibits excellent properties for odontoblast differentiation in vitro.

It is important to note that uncured monomers released from bonding agents and composite resins can leach into dental pulp tissue [37]. Some of these monomers, such as 10-methacryloyloxydecyl dihydrogen phosphate (MDP), 2,2-bis [4-(3-methylpropene acyloxy-2-hydroxypropoxy) phenyl] propane (Bis-GMA), and triethylene glycol dimethacrylate (TEGDMA), exhibit inhibitory effects on the viability and proliferation of MDPC-23 cells in vitro [38], indicating potential cytotoxicity. The chemical structure of the monomer has a significant effect on its cytotoxicity. Monomers with a high molecular weight, such as bis-GMA, are more cytotoxic than low-molecular-weight monomers, such as HEMA, as shown in a previous study [39]. Similarly, studies have shown that MDP exerts lesser inhibitory effects on cell proliferation than Bis-GMA and TEGDMA, possibly because of its hydrophilic nature, which allows it to bind easily to the hydrophilic components on the outer cell membrane without causing detrimental changes to cell morphology or proliferation [38]. CMET is the calcium salt of 4-MET and exists as a monomer and dimer in the bonding agent [25]. Therefore, it has a low molecular weight and is hydrophilic. The low cytotoxicity of CMET can be attributed to these characteristics.

The potential hydrogen of dental materials is another factor that affects biocompatibility and cytotoxicity. Dental adhesive systems and Ca(OH)_2_-based materials used in dentistry have pH values that are unusually low and high, respectively. This fact can disrupt the pH homeostasis of cells and induce apoptosis through various mechanisms, including control of mitochondrial reactive oxygen (ROS) species production, resulting in cell cycle arrest followed by apoptosis [40]. However, CMET neutralizes the strong acidity of 4-MET [27], suggesting a low cytotoxicity. Furthermore, the low concentration of CMET promoted the proliferation of odontoblast-like cells. 

Quantitative real-time RT-PCR and CPC quantification were conducted to investigate the effects of BA bond on MDPC-23 cell differentiation in this study. The BA bond significantly upregulated the mRNA expression of the dentinogenesis-related markers DSPP and DMP-1 and promoted odontogenic differentiation and mineralization [41,42,43,44,45]. ALP activity is an essential indicator of osteogenic/odontogenic differentiation and is involved in calcified reparative dentin formation. This study showed that the ALP activity in the 0.5% BA bond group was significantly higher than that in the BA (−) and SC groups. These results are attributed to the bioactivity of CMET. In contrast, no effects on cell differentiation were observed in other groups due to the bioactive components’ absence.

Ca(OH)_2_ forms reparative dentin by dissociating calcium and hydroxyl ions upon dissolution [46]. Thus, also concerning CMET, calcium ions can play an essential role in cell adhesion, proliferation, and differentiation and then promote the formation and calcification of dentin bridges. Our previous study demonstrated that CMET, a calcium salt of 4-MET, can release calcium ions that can react with phosphate ions in the tissue fluid, leading to hydroxyapatite formation in vitro [25]. The results of this study revealed that the BA bond stimulated calcific nodule formation in odontoblast-like cells. Thus, calcium ions released from CMET may be crucial in calcific nodule formation in odontoblast-like cells. 

There seem to be some mechanisms of intracellular signaling transduction concerning CMET-induced odontoblast differentiation. There is potential for the activation of some signaling pathways by the intermediary of calcium ionotropic membrane receptors [47] for calcium ions released from CMET, as well as the involvement of some integrin subunits existing on the cell membrane [48]. We previously found that CMET significantly enhances mRNA expression of integrin subunits, ITGA3, ITGA5, ITGB1, and ITGB5, and the exposure of CMET to p38 mitogen-activated protein kinases (MAPK) inhibitor significantly decreases the ALP activity and mineralization of MDPC-23 cells [27]. In a study using human dental pulp stem cells (DPSCs), the inhibition of p38 and the calcium ionotropic membrane receptor (JNK) signaling pathway completely suppressed the accelerated mineralization due to CMET in hDPSCs [28]. Moreover, inhibition of the NF-κB signaling pathway drastically diminished the CMET-induced differentiation of hDPSCs into odontoblasts [28]. Therefore, it is suggested that some signaling pathways, such as p38, JNK, and NF-κB, through the receptors, are involved in CMET-induced odontoblast differentiation.

The development of such a multifunctional innovative material will contribute to caries prevention, repair of infected decalcified dentin in sealed restorations, and dentin regeneration in deep-seated caries. It will also change the mainstream dental caries treatment completely from a “drilling and filling treatment” to “self-repair treatment without drilling.” However, this study has certain limitations regarding the CMET elution method, the 2D cell-culture system, the experimental period, etc. Further studies that account for the functions of the BA bond, such as its mechanical properties, dentin-bonding properties, antibacterial activity, and remineralization activity in vitro and in vivo, would provide substantial evidence for its potential in clinical settings.

## 5. Conclusions

The present study demonstrated that the BA bond, a novel universal bond containing the bioactive monomer CMET, promotes proliferation, differentiation at a 0.5% concentration, and mineralization in a dose-dependent manner. Conversely, the other two bonds which do not contain CMET lacked these beneficial effects. Hence, the BA bond with CMET exhibits excellent biocompatibility and is suggested to have immense potential for dentin regeneration as a multifunctional adhesive material.

## Figures and Tables

**Table 1:** Components and chemical formulations of the bonding agents.

Bonding Agent	Components	Chemical Formulation
Bioactive universal bond (Code: DP-023) (BA bond) + functional brush	Bonding agent	MDP, HEMA, ethanol, silane compound, others
	Functional brush	CMET, others
Scotchbond Universal Plus adhesive (SC, 3M ESPE, USA)	Bonding agent	MDP, HEMA, dimethacrylates, vitrebond copolymer, filler, photoinitiator, ethanol, water, silane

CMET, calcium 4-methacryloxyethyl trimellitic acid; HEMA, 2-hydroxyethyl methacrylate; MDP, 10-methacryloyloxydecyl dihydrogen phosphate.

**Table 2:** Primer sequences and PCR conditions.

Gene Name	Forward (3′ to 5′)	Backward (5′ to 3′)	Fragment Size (bp)	Tm
DSPP	TCAATGGCGGGTGCTTTAGA	TGCTCACTGCACAACATGAAGA	111	62
DMP-1	CGTTCCTCTGGGGGCTGTCC	CCGGGATCATCGCTCTGCATC	577	60
β-Actin	AACCCTAAGGCCAACAGTGAAAAG	TCATGAAGTAGTCTGTGAGGT	241	53

DMP-1, dentin matrix protein-1; DSPP, dentin sialophosphoprotein.

## Data Availability

The data supporting the findings of this study are available from the corresponding author upon reasonable request.
